# The Role of Proteomics in Bacterial Response to Antibiotics

**DOI:** 10.3390/ph13090214

**Published:** 2020-08-27

**Authors:** Foteini Tsakou, Rosa Jersie-Christensen, Håvard Jenssen, Biljana Mojsoska

**Affiliations:** Department of Science and Environment, Roskilde University, 4000 Roskilde, Denmark; ftsakou@ruc.dk (F.T.); rrj@ruc.dk (R.J.-C.); jenssen@ruc.dk (H.J.)

**Keywords:** proteomics, antibiotics, antibiotic resistance, antibiotic tolerance, multi-drug resistant bacteria, pathogens

## Abstract

For many years, we have tried to use antibiotics to eliminate the persistence of pathogenic bacteria. However, these infectious agents can recover from antibiotic challenges through various mechanisms, including drug resistance and antibiotic tolerance, and continue to pose a global threat to human health. To design more efficient treatments against bacterial infections, detailed knowledge about the bacterial response to the commonly used antibiotics is required. Proteomics is a well-suited and powerful tool to study molecular response to antimicrobial compounds. Bacterial response profiling from system-level investigations could increase our understanding of bacterial adaptation, the mechanisms behind antibiotic resistance and tolerance development. In this review, we aim to provide an overview of bacterial response to the most common antibiotics with a focus on the identification of dynamic proteome responses, and through published studies, to elucidate the formation mechanism of resistant and tolerant bacterial phenotypes.

## 1. Introduction

Successful treatment and prevention of bacterial infections in clinics meet several challenges. Some are posed by the ability of pathogens to counteract the activity of antibiotics by developing resistance and others come from the dynamic response of bacteria to different stress factors that include biofilm formation or development of persister or antibiotic tolerant phenotypes.

Antimicrobial resistance (AMR) is naturally acquired in microorganisms including bacteria, viruses, fungi, and parasites through mutation or uptake of genetic material. Today AMR is one of the main global health threats. The most urgent problem is AMR in bacteria, and over the years it has been observed that bacteria have developed resistance to every single antibiotic that has come to the market [1]. According to the Center for Disease Control and Prevention, antibiotic-resistant infections affect more than 2.8 million people annually, resulting in at least 35,000 deaths [2]. In Europe, the infections and deaths caused by multidrug-resistant bacteria are at a high rate, with estimated 670,000 infections and 33,000 deaths in 2015 [3]. Moreover, it is predicted that deaths due to AMR will increase by up to 10 million per year by 2050. This number is higher than the predicted burden of, e.g., cancer and diabetes combined [4]. Due to the misuse of antibiotics, the development and transfer of resistance mechanisms has given rise to multi-drug resistant (MDR) bacteria. In parallel with the antibiotic resistance development, microbial biofilms provide a great platform for an even higher frequency of mutation, thus introducing a greater opportunity for resistance to accumulate and spread [5]. As victims of their success, despite the number of studies that demonstrate the effectiveness of antibiotics, much knowledge on the impact of antibiotics on the overall biological network in bacteria on a system-level is lacking. In this review, we will outline 1) pathogenic bacteria, 2) antibiotic resistance and the persister phenotype as a common cause of unsuccessful treatment of bacterial infections, 3) the effectiveness of common antibiotic classes, and 4) application of proteomics as a powerful tool for the discovery of differentially expressed proteins in bacteria. We will summarize previous studies and underline the different proteomic approaches that can be used to investigate bacterial responses to antibiotics.

### 1.1. Multidrug Resistance (MDR), Priority Pathogens and Antibiotic Tolerant Persister Phenotype

Antibiotic resistance in bacteria is a multifaceted issue. Bacteria can naturally mutate to develop resistance mechanisms to antibiotics. Additionally, bacteria can transfer resistance genes across species through horizontal gene transfer [6]. The resistance mechanisms can be broadly categorized into three basic patterns: the prevention of drug accumulation, modification of cell target, and inactivation of antibiotics [7]. Each mechanism acts differently depending on the bacterial species it originates from. For instance, rapid adaptation of *P. aeruginosa* to environmental stresses has been attributed to (among other characteristics) the high percentage of genome encoding for two-component system elements, including numerous atypical kinases, which provide stricter control over regulatory responses to environmental triggers [8]. Some examples of two-component systems that have key roles in the regulation of virulence and antimicrobial resistance include PhoP-PhoQ, pmrA-pmrB, and CprRS involved in polymyxin B and antimicrobial peptides (AMPs) resistance, and GacS-GAcA and PprB-PprA involved in aminoglycoside resistance [2,9]. The pmr operon controlled by the last two of these two-component systems mediates antimicrobial peptide resistance. Besides, extracellular DNA (eDNA), which is an important factor in response to AMP-caused stress, is also contributing to the protection of the outer membrane from antimicrobial peptides-caused damage through activation of the PhoPQ and PmrAB systems and indirect regulation of PA4773-PA4775 genes. This activation leads to the production of spermidine, which interacts electrostatically with the negative charges of the O-antigen, preventing the peptides from binding instead [10]. Decreased susceptibility to antibiotics is also achieved through stabilization of the inner lipopolysaccharide core. Two genes (*wapP* and *wapQ*) encoding for enzymes that phosphorylate the inner core heptoses (Hep) decrease antibiotic and detergent permeability and have a crucial role in cell viability [10]. Active drug efflux through efflux pumps is also a very common resistance mechanism in *Pseudomonas* species, since this bacterium employs more than twelve pumps to help cope with antibiotic stress [11]. OprM is a major protein component of active efflux that is involved in the activity of several different pumps. In *P. aeruginosa*, OprM undergoes a post-translational modification by N-terminal palmitoylation, the role of which is not yet clear, and investigation of the different enzymes involved in this process could provide an insight regarding this alteration and could also serve as a future therapeutic target to combat antibiotic resistance [11].

In 2017, the World Health Organization (WHO) published a list of 12 priority pathogens that urgently need development of effective antibiotics. All the pathogens presented by the WHO are also multi-drug resistant, meaning that they are resistant to more than one antibiotic class. The bacterial species are classified into three groups, depending on antimicrobial resistance, ranging from species of critical (three species), through high (six species) and down to medium (three species) importance (Table 1) (WHO 2017). The overview emphasizes the importance of accelerating drug discovery pipelines and/or re-thinking of the way we use the antibiotics that we have at our disposal. The list contains nine Gram-negative and three Gram-positive bacteria, which emphasizes that Gram-negative bacteria, in general, are a broader cause for concern, though Gram-positive bacteria still result in the largest economical burdens in the healthcare and intensive care unit. Specifically, the most challenging infections are caused by methicillin-resistant *Staphylococcus aureus* (MRSA), vancomycin-intermediate *S. aureus* (VISA), vancomycin-resistant *S. aureus* (VRSA), and vancomycin-resistant *enterococci* (VRE). The emergence of such strains also leads to an increase in the cost of treating infections caused by Gram-positive bacteria [12]. The pathogen list contains also the ESKAPE pathogens (*Enterococcus faecium*, *S. aureus*, *Klebsiella pneumoniae*, *Aacinetobacter baumannii*, *P. aeruginosa,* and *Enterobacter* species) which are considered to be the leading cause of nosocomial infections [13] with the highest risk of mortality [14].

Besides the high prevalence of the resistance phenotype among bacteria, several studies have reported on another phenotype that potentially contributes to the inefficiency of antibiotic treatments [15,16,17]. This phenotype is described as a persister phenotype where antibiotic tolerant persister subpopulations do not grow in presence of antibiotics, but remain dormant during the antibiotic exposure and have the ability to resuscitate once the antibiotic is removed [18]. To decipher the mechanism of persister cells formation, studies have reported on the role of possible genetic variants [19], toxin-antitoxin systems [20], metabolic downregulation via transcriptome analysis [21], reactive oxygen species induction mechanisms [22], arrested protein synthesis [23], and others. Since the mechanism of this phenotype is not well-understood, efforts to study the process of persister cells formation need to investigate the bacterial response on a system level. Especially, proteomic studies could significantly contribute to this area, since the persister phenotype is not a result of a genetic change.

### 1.2. Proteomics by Mass Spectrometry as A Tool and Common Data Acquisition Strategies

Mass spectrometry is a technique that has been used for the identification of peptides since the mid-1980s. However, it was not until the mid-1990s, with full genome sequencing techniques and advancements in computer technology, that proteomics by mass spectrometry was born. The rapid development of mass spectrometers and software since then has made this a very attractive method for analyzing biomolecules. Proteomics is a method for studying more complex protein mixtures, such as bacterial lysates or clinical tissue samples containing several thousands of proteins. Since no mass spectrometer can gain useful information on direct injection of this very complex matrix, there is a need for protein or peptide separation before introduction to the mass spectrometer. There are several methods of reducing sample complexity. One of the early methods was the separation of proteins by 2-dimensional gel electrophoresis (2DE) followed by in-gel trypsin digestion of individual protein spots on the gel, and subsequently conducting mass spectrometry analysis on a Matrix-Assisted Laser Desorption Ionization Time-of-Flight (MALDI-TOF) instrument. This method was quickly followed by SDS-PAGE gel separation of proteins combined with in-gel trypsin digestion of protein bands subjected to liquid chromatography (LC) separation, typically reverse phase, directly connected with an electrospray ionization (ESI) instrument. Because of the rapid development in the mass spectrometry field, and especially within the orbitrap technology, the instruments are now fast enough to identify several thousands of proteins in few hours without prior gel separation and can now be conducted by injecting trypsin-digested whole lysates. Post-translational modifications (PTMs) of proteins can often be of most significant biological relevance, but since these are often in low abundance, they cannot be analyzed with a regular whole lysate mass spectrometry (MS) analysis. This type of analysis typically requires an enrichment step of the investigated modification. Protocols for the enrichment of phosphorylation, acetylation, methylation, and many others are already well established and for further information on PTM studies and methodologies in bacteria we recommend the review article by Macek et. al. [24]. Identification of proteins is very important but more important, in a biological context, is the quantification of protein abundances between different biological states. This knowledge can lead to a greater understanding of the biomolecular mechanism of the investigated topic. Mainly, there are two different protein quantification strategies, label-free and label based. Both MS-based approaches have different limitations and the choice largely depends on several factors, including the research question. Using these two approaches to study bacterial proteome has resulted in different number of confidently identified proteins, 178 and 421, label-based, iTRAQ, and label-free, respectively [25]. Within the labeling methods, there are several different techniques. These include metabolic labeling, such as stable isotope labeling using amino acids in cell culture (SILAC) [26] or bacterial growth media [27], and chemical labeling on peptide level, such as isobaric tags for relative and absolute quantitation (iTRAQ) [28] or tandem mass tags (TMT) [29]. Other more targeted labeling techniques, such as pulsed labeling, can be used to study protein turnover, since it will only label proteins synthesized after, for example, antibiotic treatment. There are many different mass spectrometry methods, but most proteomics studies are still using data-dependent acquisition (DDA), which means that the data acquired within the run are dependent on the data obtained on-the-fly. This method will typically favor the more abundant peptides. Data independent acquisition (DIA) does not rely on the data acquired by the mass spectrometer and does not depend on peptide abundance, but this will generate very complex data analysis and interpretation. When the samples have been analyzed by LC-MS/MS, there is typically more than one software that can process the data. Several search software is available such as MaxQuant, Mascot, Byonic, Skyline, and PEAKS. After the processing of the raw data comes the interpretation of the obtained data. This process can vary greatly among different labs and it also typically depends on the instrument vendor. Perseus is compatible with MaxQuant and Proteome Discoverer can be implemented with several of the above-mentioned search software.

### 1.3. Applications of Proteomic Data Acquisition in Studies of Bacteria

Innovative ‘omics’ technologies, such as functional genomics, transcriptomics, metabolomics, and proteomics, generate a plethora of material that assists progress made in clinical microbiology. These technologies have increased our overall understanding of the complexity of microbial systems. Since many genome databases offer great coverage of completely sequenced genomes, some domains provide the total protein sequences and their function (www.uniprot.org) identified in multiple bacterial species. We can use this information to investigate differential protein levels in the presence of different stress factors in bacteria, as well as identify and quantify protein modifications that happened during protein synthesis or after protein translation. This is especially important since other omics technologies, such as genomics and transcriptomics, cannot capture post-translational changes in proteins. Proteomic analyses have a wide range of applications. Examples include investigating different protein levels in resistant [30] and drug-tolerant bacterial phenotypes [31] (Table 2), as well as reports on the bacterial adaptation to different growing conditions and bacterial catabolism [32], biomarker discovery [33], and pathogen-host cell interactions [34,35]. In several other studies, different proteome analysis approaches have been employed to study the bacterial response to commonly used antibiotics such as ciprofloxacin [36,37,38,39,40], tobramycin [41,42], colistin [43,44,45], polymyxin B [46], daptomycin [47,48], and silver nanoparticles [49] (Table 2). In addition, many biological processes are regulated by post-translational modifications (PTMs). Advances in proteomic analysis have allowed for the identification of around 250 protein modifications in bacteria [50] that happen as post-translational modifications or during the process of translation (co-translational). Some include modification of existing amino acids along the polypeptide chain (deamination, elimination, etc.), addition of chemical groups or multiple peptides (phosphorylation, acetylation, ubiquitination, etc.), or sequence proteolysis where existing amino acids are removed from the polypeptide chain [51]. Protein modifications can greatly alter the protein behavior and therefore these modifications can be used as markers for change in protein-protein interaction, ligand binding, and the overall change in protein function. Specifically, enzymes that are involved in such processes are possible targets for antimicrobial drug development against pathogenic bacteria. All of these have been extensively described [24]. To aid protein identification and understanding of the modified protein role in bacterial processes especially in the field of antibiotic resistance, there are several databases available, one of which is the Comprehensive Antibiotic Resistance Database (CARD, https://card.mcmaster.ca/). Examples of applications of proteomic analysis in research studies investigating *P. aeruginosa, E. coli,* and *Mycobacterium tuberculosis* will be briefly described.

*P. aeruginosa* is an opportunistic pathogen that chronically infects the lungs of cystic fibrosis patients and individuals suffering from obstructive pulmonary disease and hospital-acquired pneumonia [52,53,54]. With a genome of 6.3-Mbp, according to the UniProt database, there are around 5570 predicted Open Reading Frames identified in *P. aeruginosa* reference strain PAO1 (https://www.uniprot.org/proteomes/UP000005565). Besides, protein modifications in this bacteria and their role in various biological processes, such as translation, central metabolism, amino acid synthesis, virulence, and resistance, have been well described [51,55]. Most recently, proteomic analysis has been used to dissect the in vivo proteome of *P. aeruginosa* in airways of cystic fibrosis patients [56]. Lysine acetylation, a type of post-translational modification, has also been characterized in *P. aeruginosa.* Identification of 320 acetylated proteins provided information on the subcellular localization of the modified components and the number of the modifies sites. The altered proteins had roles in some of the main metabolic pathways, lipopolysaccharide biosynthesis, and virulence [57].

*Mycobacterium tuberculosis*, which remains a health concern, encodes around 3993 proteins based on UniProt database (https://www.uniprot.org/proteomes/UP000001584). Label-free methodologies and data-independent acquisition strategies have been used by Schubert and coworkers to investigate the proteome response during dormancy and understanding the proteomic profile in this physical state to gain insight into the metabolic adaptation. In this study, the authors have successfully covered more than 2,000 proteins and estimated the total cellular protein concentrations in this pathogen [40].

In *E. coli* reference strain K12, there are currently around 4,391 identified proteins (https://www.uniprot.org/proteomes/UP000000625). Mass spectrometry-based proteomics has again enabled measuring cellular protein concentrations in *E. coli* under different experimental conditions and exploring the molecular network in this bacteria [58]. In two other studies, a large number of proteins have been identified as differentially expressed or enriched (710 proteins) in *E. coli* antibiotic tolerant populations [59,60] (Table 2).

## 2. The Role of Proteomic Analysis in Generating New Insight about The Mechanism of Action of Antibiotics and Antibiotic Resistance

**Figure 1:**
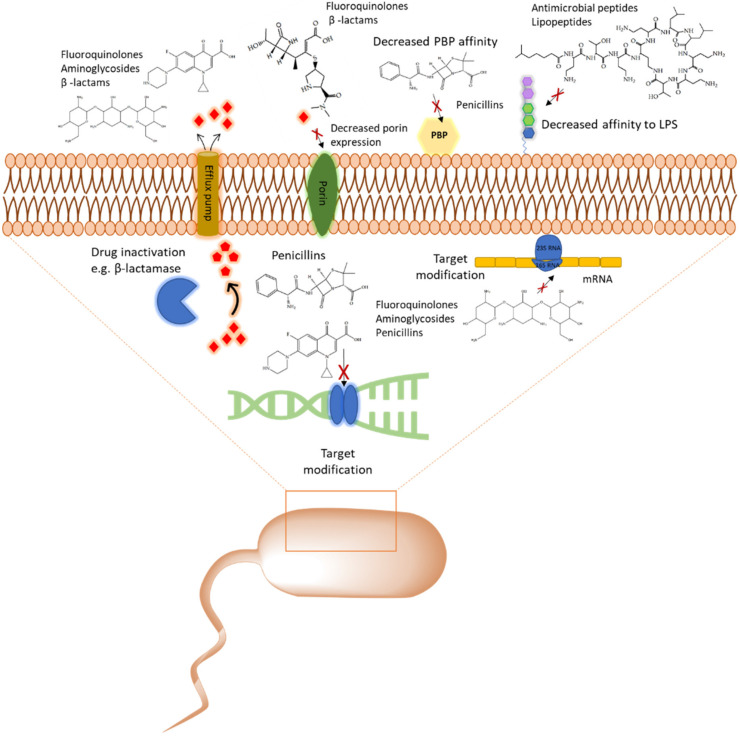
Overview of the common antibiotic resistance mechanism in bacteria. Molecular mechanisms of antibiotic resistance that includes target modification, drug inactivation, decreased affinity to lipopolysaccharides (LPS) and penicillin binding protein (PBP), and expression of porins and efflux pumps are shown.

With the emergence of antibiotic resistance genes among pathogenic bacteria, proteomic analyses are pivotal in the assessment of the dynamic changes of whole protein expression on a system level. When studying the proteome of pathogenic bacteria there is a high interest to obtain a quantitative view of the differentially expressed proteins in different treatment conditions. Using targeted proteomics one can monitor resistance development and behavior and understand the role of cellular processes when pathogenic bacteria are challenged with antibiotics. In this section, several classes of antibiotics, their mode of action, and studies that report on using proteomic approaches to further explore the bacterial response to these antibiotics will be reviewed. A summary of mechanism of antibiotic resistance is presented in Figure 1 and a table that summarizes different antibiotics, experimental conditions, and methods and analysis approaches used from multiple studies, is also included (Table 2).

### 2.1. Cell Wall Synthesis Inhibitors

**Figure 2:**
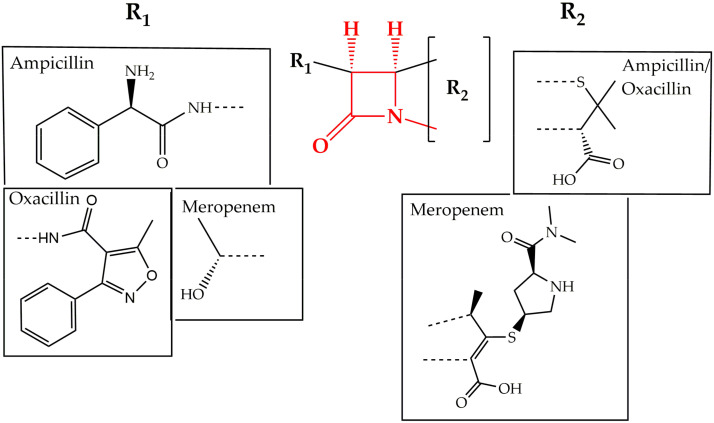
Chemical structure of conserved ring structure in β-lactam antibiotics and side-chain functionalities (R_1_ and R_2_) of three β-lactam antibiotics, ampicillin, oxacillin, and meropenem. Dashed lines represent the connecting bonds to the β-lactam ring (**red**).

Cell wall synthesis inhibitors are a class of antibiotics with the widest use, with Penicillin class (e.g., penicillin G, ampicillin, and oxacillin) being the best known and oldest precursor. Cephalosporins, Cephamycins, Monobactams, and Carbapenems (e.g., meropenem) comprise some of the derivative sub-classes all sharing a β-lactam ring (Figure 2) as a common structural feature [73]. The primary mode of action of this class of antibiotics is inhibition of bacterial cell wall synthesis through binding to an active serine site of penicillin-binding proteins and rendering the enzymes inactive [74] (Figure 1). Several different penicillin-binding proteins can be simultaneously targeted in a single organism affecting different cellular functions [75]. In this way, the enzymes are prevented from catalyzing both the synthesis and cross-linking of peptidoglycan which is essential to achieve a rigid structure. The major lethal consequence is a loss of cell wall integrity and cell death due to osmotic imbalance and/or digestion of the existing cell wall by peptidoglycan hydrolases [76]. However, except for the enzymes that are bound by the antibiotic, more complexes involved in peptidoglycan synthesis are also indirectly affected. This is because of the gradual shortage of peptidoglycan precursors, which makes the cell unable to keep up with the required peptidoglycan synthesis pace to preserve a rigid and efficiently protective wall structure [77].

Studies on the bacterial response to ampicillin have been done in *E. coli*. Exposure to sub-MIC of ampicillin together with tetracycline has been used to characterize and compare outer membrane proteome changes in *E. coli* K12 strain [78]. With eight protein being differentially regulated over ampicillin exposure, ampicillin induced the upregulation of porins OmpC, OmpW, and Tsx, the efflux pump subunit TolC, the usher protein FimD, the Omp assembly factor BamD and the hypothetical lipoprotein YfiO. In this study decreased expression was observed only for the outer membrane protein BamC [78]. In addition, specific protein compounds were associated with outer membrane vesicles-related β-lactam resistance and were also able to confer resistance to susceptible strains in a dose-dependent manner. These proteins included OmpC, OmpF, OmpW, Tolc, and Bcl1. Proteins linked to resistance to different antimicrobial agents (both antibiotics and peptides) were also identified. The suggested resistance mechanism was that the antibiotic could be transferred at a high rate through the overexpressed porins to the lumen of the outer membrane vesicles and get hydrolyzed by β -lactamase enzyme (Figure 1) before being able to act on the cell wall [63] (Table 2). Mass spectrometry analysis and the Sequential window acquisition of all theoretical mass spectra (SWATH) methods were used to compare the adaptive changes between three different, ciprofloxacin-resistant, carbapenemase-producing *Enterobacteriaceae* (CPE) strains under meropenem and ciprofloxacin stress. Each antibiotic had caused different proteome alterations and the difference in protein expression has been reported to be more intense in the meropenem-treated cells. Overexpression of the histone-like protein HU, the GroEL/GroES chaperone complex, and the nucleotide exchanging factor Grpe was observed in all carbapenemase-producing strains with the most striking difference in those producing New Delhi metallo-β -lactamase, indicating that DNA and protein stability could be the main factors to be enhanced to increase bacterial fitness. On the contrary, ciprofloxacin only seemed to affect the levels of the outer membrane protein OmpA [64] (Table 2). Multidrug-resistant sequence type 131 strains have also been targeted through proteomics, in extended-spectrum β lactamase (ESBL)-producing *E. coli* strains. Five proteins in total were identified and connected to this resistance phenotype, including YahO, YjbJ, YnfD, HdeA, and soluble cytochrome b562, all having an amino acid replaced which was specific for the ST131 sequence type (Table 2). Through bioinformatics, it was also possible to provide a prediction on how these proteins might interact to contribute to spreading and maintaining the infection in the human intestinal tract. It was suggested that some of the proteins enhance tolerance of the bacteria to gastric acid and some of them prolong the infection through biofilm formation in the intestinal tract [62]. Proteins involved in protein biosynthesis and regulation have also been quantified as the most abundant in clinical strains of *E. coli* (C538 and C580), producing β-lactamases CMY-2 and TEM-52 [72] (Table 2). In addition, the MRSA and Methicillin-susceptible *S. aureus* (MSSA) response to oxacillin, another β-lactam antibiotic, has been investigated using label-free quantitative proteomic strategy. In this study, commonly and differentially expressed proteins had been identified offering complete overview of the antibiotic response in resistant and susceptible bacterial strains [69] (Table 2).

### 2.2. Inhibitors of Protein Translation

#### 2.2.1. Aminoglycosides

**Figure 3:**
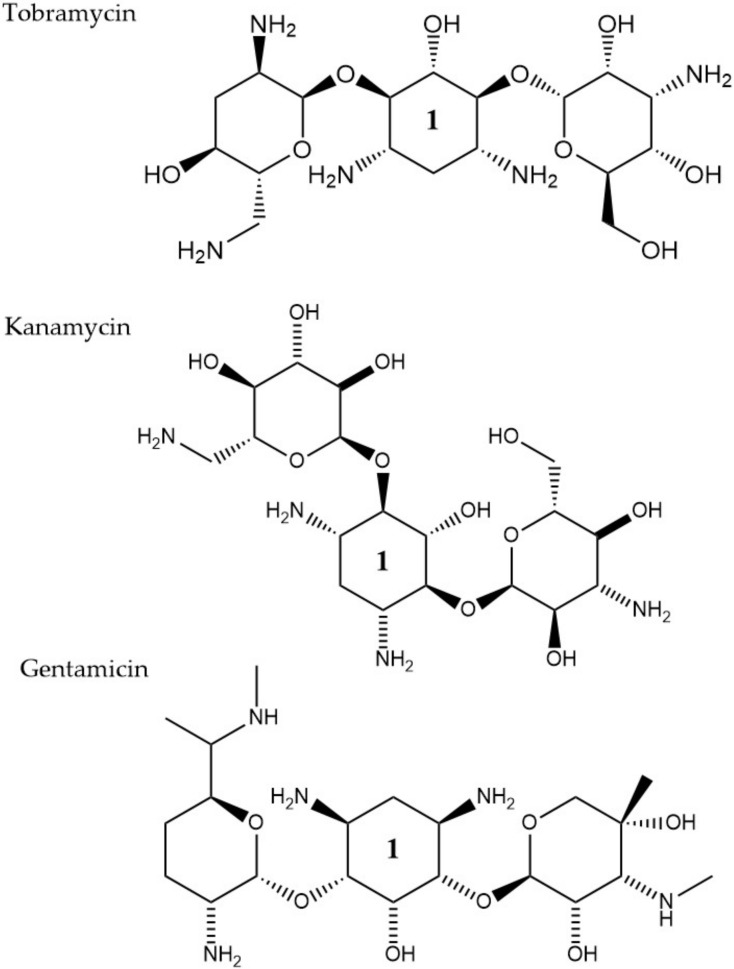
The chemical structures of three aminoglycoside compound. r tobramycin, kanamycin, and gentamicin are shown. All three compounds chare similar ring number 1 indicated across the structures.

Aminoglycosides, along with tetracyclines and macrolides, are inhibitors of protein translation and represent (Figure 3) a class of antibiotics with a broad activity spectrum that are being used to target infections from both Gram-negative and Gram-positive bacteria, but most commonly those caused by *Enterobacteria*, such as *E. coli* [79]. Aminoglycosides exert their activity by inserting themselves into the bacterial cell. This process consists of three consecutive phases [29]. Initially, because of their polycationic nature, aminoglycosides bind through electrostatic interactions to the negatively charged portions of the bacterial membranes [80]. The main bacterial components involved in this interaction are phospholipids and lipopolysaccharides, and teichoic acids in Gram-negative and Gram-positive bacteria, respectively [81]. Displacement of divalent cations is facilitating this interaction with the outer membrane leading to its destabilization. The increased permeability of the membrane facilitates a self-promoted uptake of the antibiotic agents into the periplasmic space [82]. During the 2nd phase, insertion into the cytoplasm is achieved through an energy-dependent manner, that takes advantage of the electron transport chain and membrane potential difference, enabling the insertion of only a few molecules [83]. Interaction of aminoglycosides with specific nucleotides of the 16S bacterial ribosomal RNA creates a barrier to protein synthesis [84]. The outcome is either halting translation or triggering production of mistranslated proteins that can further enhance the drug uptake into the cell by creating membrane pores. The last stage involves higher accumulation levels of the antibiotic in the cell, and hence, a faster pace of translation inhibition as well as mistranslation, explaining the fast and concentration-dependent bacterial killing observed in this particular antibiotic class [85]. Tobramycin, isolated from *S. tenebrarius,* is a widely used aminoglycoside antibiotic in the clinics and since its first introduction in 1976 [79]. Although the overall class has no strong activity against *P. aeruginosa*, tobramycin was the most potent agent against it, following amikacin [86]. It has been extensively used alone or in combination with other antibiotics, for treating lung infections in cystic fibrosis patients [87]. Tobramycin, as most aminoglycosides, is a fast-acting bactericidal agent and its action is highly affected by its concentration [86]. Although it has been poorly studied, there is evidence that tobramycin acts through multiple mechanisms. Pharmacodynamic model proposing a dual mechanism of tobramycin against *P. aeruginosa* with two killing modes: a delayed mode accounting for the effect on protein synthesis (requiring lower concentration) and a more rapid mode corresponding to membrane disruption (requiring higher dosage) has been suggested [88]. Finally, the interaction of tobramycin with the outer membrane can facilitate the entry of additional antibiotics when used in combination with, e.g., β-lactams [89].

LC-MS analysis has further been employed to address the hypothesis that tobramycin could facilitate the eradication of *P. aeruginosa* infections in cystic fibrosis, through decreasing levels of virulence determinants in secreted outer membrane vesicles. Total proteome analysis of these vesicles revealed 757 proteins, with 66 core proteins also detected in four earlier proteomic studies [90,91,92,93] and 120 proteins were conserved in four *P. aeruginosa* strains, including two CF clinical ones. Many of the conserved proteins are associated with resistance and virulence, such as efflux pump elements MexA and MexB. After exposure to tobramycin, 165 proteins were downregulated and 17 were up-regulated in the isolated outer membrane vesicles (Table 2). One of the proteins that were significantly decreased was the virulence factor AprA. This factor is a protease, assumed to indirectly promote dehydration of the airways by inhibiting the secretion of Phe508del-CFTR-Cl^−^ by the epithelial cells of the host and as a result, the clearance of the infection becomes more challenging. The addition of tobramycin- exposed and mutant ΔaprA outer membrane vesicles to bronchial epithelial cells from cystic fibrosis patients led to a decrease in the inhibitory effect of *P. aeruginosa* on Phe508del-CFTR-Cl- secretion. Finally, significantly lower expression levels were also reported for the virulence factors AlpA/D/E, after tobramycin treatment. All the findings together suggest that tobramycin could improve lung function in cystic fibrosis patients [42]. Adaptive resistance of *P. aeruginosa* to tobramycin under planktonic conditions has also been investigated through proteomics analysis [41]. The study focused on the evaluation of any proteome changes both, after treatment with a range of sub-MIC concentrations and over a time-course exposure using a fixed tobramycin concentration. Different protein alterations appeared to be clustering together in either higher or lower concentrations. Higher antibiotic dosages induced upregulation of heat shock proteins and proteases, while overexpression of proteins involved in amino acid metabolic/catabolic pathways was observed in treatment using lower drug concentrations. The lowest tobramycin concentration and the highest exposure time induced the wider range of proteome changes among all tested conditions. The most highly up-regulated protein was a heat shock protein (IbpA) that was found to assist cells to resist the effects of the antibiotic by acting together with other proteases, heat shock, and chaperone proteins [41]. In other studies, several proteins of outer membrane vesicles were identified to have a system-level role in antibiotic resistance to other types of aminoglycosides, such as streptomycin [94] and gentamicin [71] (Table 2). Resistance to Kanamycin, which is another aminoglycoside (Figure 3), through outer membrane proteome modification profiling, was also investigated through mass spectrometry and Western blot. Here, an increase in the levels of TolC, Tsx, and OstA and a decrease in OmpA, FadL OmpW, and MipA, was observed leading the authors to suggest that MipA was a new protein linked to aminoglycoside resistance [68] (Table 2). Targeted proteomic approaches were employed to study protein pattern alterations that accompany interaction between the two-component system elements CpxA and ArcA in *E. coli* MG1655 cells when challenged with gentamicin. Here, differentially expressed proteins in wild type and cpxAR mutant strain have been identified including proteins related to metabolism (FruA, FruK, and IlbB) and in stress response (IbpB, IbpA, RpoH, YgiQ, YceA, YncD, YrbL, and DeaD) [95].

#### 2.2.2. Macrolides

**Figure 4:**
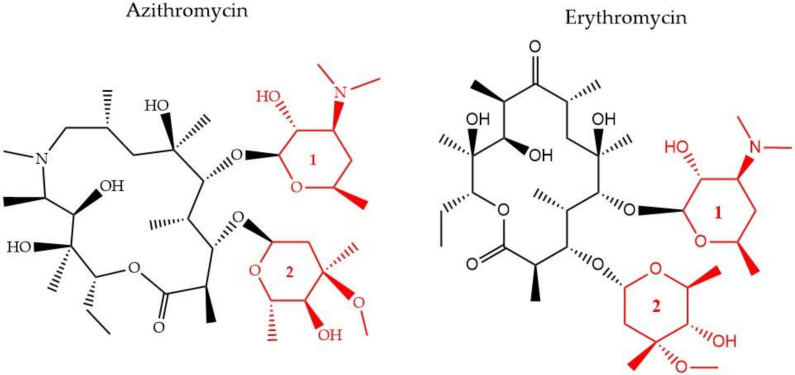
The chemical structure of two antibiotics, azithromycin, and erythromycin that belong to the macrolide class are shown. Conserved ring structures of sugars 1 and 2 (**red**) and varied lactone ring are shown.

There have been three generations of macrolides developed since 1952. The first generation consists of natural components, while the later generations are semi-synthetic and have a wide activity spectrum [96]. The main structural characteristic of this class is a 12–16-member lactone ring with one or more sugars attached (Figure 4). For years, the main mode of action was suggested to be inhibition of protein synthesis merely through association with the 50S ribosomal subunit and obstructing the nascent peptide exit tunnel [97]. Although the precise mode of action remains unclear, several studies over the years have provided insight into new aspects of macrolides’ impact on protein translation. It is now known that macrolides, instead of inducing complete protein synthesis suppression, interfere with specific protein motifs and therefore selectively block peptide elongation [98]. Macrolides can have both bacteriostatic and bactericidal activity [99]. It has been also suggested that the major amino acid sequence responsible for inhibiting translation is Lys/Arg-X-Lys/Arg where both the positive charge and side chains play a role in this process [100].

Furthermore, it is hypothesized that besides the recognized sequence mentioned above, there are additional protein fragments, depending on each protein’s sequence, that could modify the peptide’s route in the nascent peptide exit tunnel NAPT and result in decreased efficiency of the antibiotic’s function or differential response to different macrolides [101]. One of the most common resistance mechanisms against macrolides, characterized in *S. aureus* [102], *S. pneumoniae* [103], and *E. coli* [104], is mediated through methylation of the 23S ribosomal domain by methyltransferases encoded by the erythromycin (Figure 3) ribosome methylase gene (*erm*). The modification leads to a decrease of the affinity between the binding target and the antibiotic, inhibiting their interaction [105] (Figure 1). Active efflux is another way to cope with macrolide-pressure mediated by mefA/E (M-type resistance) and msrD genes in *S. pneumoniae* [106] and *S. pyogenes* [107]. Macrolide active efflux has also been characterized in *Enterobacteriaceae* [108], as well as macrolide-inactivation by phosphorylases and esterases, encoded by ereA/B and mphA/B genes, respectively [104]. The development of resistance in *E. coli* populations after exposure to sublethal concentrations of erythromycin has been characterized by MALDI-TOF MS and protein radiolabeling. Different exposure times (43, 68, and 103 h) to the antibiotic appeared to lead to the development of different proteome profiles. The proteins that were down-regulated after the shortest exposure time frame were in contrary, up-regulated following the longest treatment. This finding was accompanied by a shift in the subcellular compartment of the up-regulated proteins from the cytoplasm to the outer/inner bacterial membrane. The result of the longest exposure was elevated expression levels in proteins linked to lipid, amino acid, carbohydrate, and polyamine metabolism together with the porin OmpC [71] (Table 2).

### 2.3. Inhibitors of DNA Synthesis

**Figure 5:**
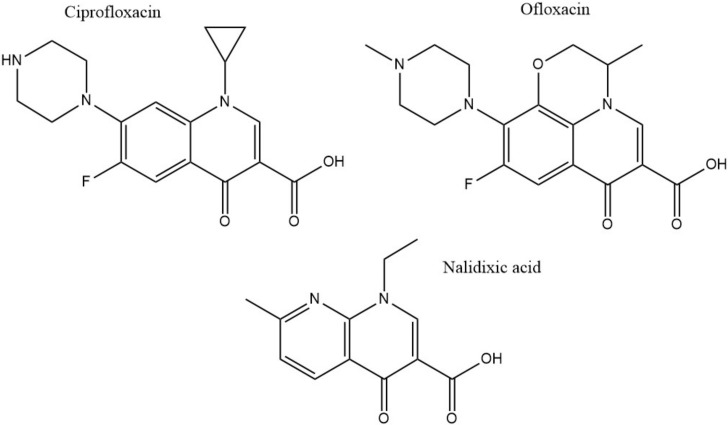
Chemical structures of three clinically used quinolone antibiotics is shown.

With the discovery of nalidixic acid in 1962, quinolone development has substantially increased and several antibiotics that share similar structural properties (Figure 5) have been developed since [109]. Fluoroquinolones are especially actively used in the treatment of bacterial infections due to their excellent activity against a wide range of Gram-negative and Gram-positive bacteria. Ciprofloxacin belongs to the broader class of quinolones and is primarily used to address infections caused by Gram-negative bacteria [43]. Among the group of fluoroquinolones, ciprofloxacin has been the most effective agent against *P. aeruginosa* [110]. This antibiotic acts by binding to DNA gyrase and IV topoisomerase in Gram-negative and Gram-positive bacteria, respectively, and inhibiting DNA replication [111]. This is achieved by stabilization of the DNA-gyrase complex during replication via interaction with the 3-oxo-4-carboxylic acid core (Figure 2) of the antibiotic [112]. The interaction with the DNA is additionally facilitated through the formation of Mg^2+^ bridges [113]. The association of the complex with ciprofloxacin results in the broken DNA strands being trapped inside the complex and unable to religate. As a consequence, RNA polymerase is additionally blocked, DNA synthesis is halted, and the bacteria die releasing cleaved DNA fragments [114]. A secondary mechanism that contributes to the bactericidal activity of ciprofloxacin is the induction of reactive oxygen species [115]. Ciprofloxacin resistance development has been studied in *P. aeruginosa.* Although genetic mutations were partly responsible for the adaptive resistance progression, proteomic analysis revealed increased phosphorylation levels of two enzymes, succinate-semialdehyde dehydrogenase (SSADH), and methylmalonate-semialdehyde dehydrogenase (MMSADH). It was hypothesized that these proteins could play a role in ATP production as a supportive mechanism to the efflux pumps. In addition, they could trigger higher nicotinamide adenine dinucleotide phosphate dehydrogenase (NADPH) production to combat the accumulation of hydroxyl radicals induced by ciprofloxacin [37].

Adaptation to ciprofloxacin exposure has also been studied in biofilm cells. Here, the outer membrane proteome modifications in *P. aeruginosa* biofilms have been identified, after treatment with benzalkonium chloride and ciprofloxacin over 12 consecutive days [38] (Table 2). Overall, only moderate modifications were reported and the expression levels of the chaperon protein GroEL, the putative tail sheath protein, and the major capsid protein were downregulated in both conditions. Since GroEL would be expected to enhance the flexibility of the bacterium to adapt to the antibiotic stress the opposite results would be anticipated. Regarding the other two proteins, although their precise function is not known it was hypothesized that they might indicate membrane degradation. Finally, ciprofloxacin induced higher levels of the probable bacteriophage protein, which is in agreement with the higher activation rates of bacteriophage genes in biofilms and could be linked to the diverse phenotypes found in a biofilm [38]. Another study looked at different mechanisms involved in a transition from low to high ciprofloxacin resistance through drug pre-exposure in *Pseudomonas*. The main physiological pathways in each type of resistance were determined using total proteomic analysis of four resistant mutants. Different regulatory pathways were involved in each of the lower resistance levels. Anaerobic respiration was adopted by the mutant with the lowest resistance and the arginine, arginine dehydrogenase, and urease pathways were up-regulated. Slightly higher resistance involved upregulation of proteins having a role in nutrient uptake, such as iron polyamine and amino acids, as well as protein translation. Regarding the mutants demonstrating the highest resistance, the MexCD-oprJ efflux pump had the highest expression levels while proteins involved in quorum sensing were the most downregulated [40]. The later serves as good supporting evidence on many studies that associate efflux pumps as a resistance mechanism to ciprofloxacin [36]. Mass spectrometry experiments confirmed that the intracellular drug concentration was unchanged between wild type cells and low resistant mutants, indicating that the differential regulation of the above-mentioned pathways could be mainly responsible for the resistance [40]. Proteomics has also been applied to selectively analyze subpopulations of *P. aeruginosa* biofilms that are tolerant to ciprofloxacin. The results showed that the immediate proteome response of antibiotic tolerant subpopulation of *P. aeruginosa* is characterized by flagellar motility, whereas the adaptive proteome response included upregulation of synthesis of purine molecules related to DNA damage response [39]. Ofloxacin, similar in structure to ciprofloxacin (Figure 2) is a second-line drug used for the treatment of multidrug-resistant tuberculosis (MDR-TB). It has the same target as ciprofloxacin which is DNA gyrase and therefore *gyrA* and *gyrB* are often targets for drug resistance. The proteomic analysis shows that there is an increased expression of 14 differentially expressed proteins in TB isolates that carry natural ofloxacin resistance and induced resistance when compared to the isolates susceptible to the drug. Follow up studies on the role of the identified proteins further revealed that few hypothetical proteins might be interfering with the ofloxacin activity by neutralizing the effect of DNA replication, transcription, repair, and recombination [67] (Table 2).

### 2.4. Cyclic Lipopeptides That are Used as Last Resort Drugs in Treatment of Bacterial Infections

**Figure 6:**
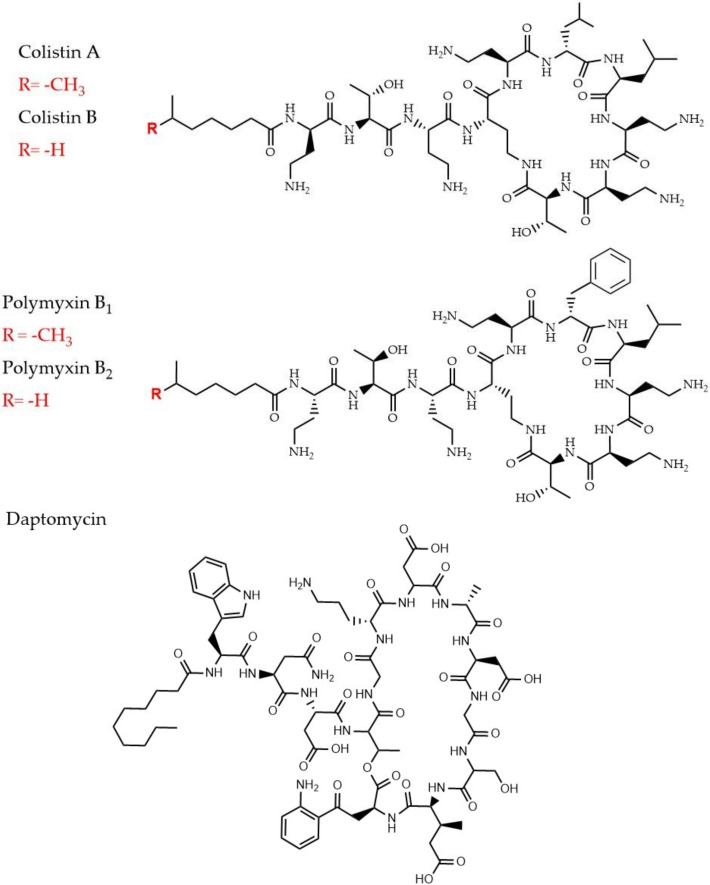
Chemical structures of cyclic lipopeptides, polymyxin (B_1_ and B_2_), colistin (A and B), and daptomycin, are shown. R=CH_3_ is polymyxin B1 and R=H is polymyxin B2. Similarly, for colistin, R=CH_3_ is colistin A and R=H is colistin B. The ring in daptomycin consist of 10 varied amino acids and the tail contains different amino acid composition along with longer carbon chain (10 carbons).

As aforementioned, antimicrobial resistance requires the development of new lines of antibiotics. If the first-line antibiotics are failing in fighting the infections the second or third-line is used instead [116]. Moreover, when all three antibiotic-lines fail, the “last resort” antibiotics are used as an alternative. Some of the last-resort antibiotics are lipopeptides that can target various organisms [117,118]. These are compounds that have a common structure where a peptide core is attached to a lipid tail (Figure 6). Lipopeptides are natural compounds produced by microorganisms such as bacteria and fungi. They possess therapeutic properties and are used in drug manufacturing [118]. In the following section known mechanism of action and proteomic analysis for two cyclic lipopeptides, polymyxin B and Colistin, will be described.

#### 2.4.1. Polymyxins and Colistin

This last-resort class of lipopeptides is used for Gram-negative bacteria infections, especially those caused by *A. baumannii, P. aeruginosa,* and *K. pneumoniae* [117,119]. There are approximately 30 different molecules of polymixins, divided into five chemical compound groups (polymixins A to E), although Polymyxin B and colistin (Polymyxin E) are the most commonly used (Figure 6) [117]. Though it is considered a drug of last resort, it should be emphasized that Colistin was banned in the 1970s, due to neurotoxicity and nephrotoxicity [120]. Hence, its reintroduction in the new millennium, due to the emergence of MDR Gram-negative bacterial strains, are not without concern. It is generally accepted that the cationic charges of polymyxin bind to negatively charged lipid A moiety of lipopolysaccharides and displace Mg^2+^ and Ca^2+^ ions which stabilize the outer membrane [121]. This displacement disrupts the membrane permeability barrier, thus allowing the uptake of polymyxin [122]). As a contradiction to this assumption, some studies have shown that cell lysis is not required for cell death in *P. aeruginosa*, which points out to the presence of other alternative mechanisms [123,124]. Additionally, intrinsic and acquired resistance mechanisms resulting in increased surface charge (decreased surface binding, Figure 1) have been described in a panel of species from *Proteus mirabilis* and *S. marcesens*, *Klebsiella, Escherichia, Enterobacter,* and *Salmonella* [117,125,126]. Adaptive regulatory mechanisms of *E. coli* to Polymyxin-B induced stress have been evaluated through a quantitative proteomics analysis (iTRAQ) [46]. The comparison of the impact of two different concentrations (low vs. high) on the protein level, revealed numerous differentially expressed proteins, mainly involved in quorum sensing, two-component systems, TCS, and fatty acid degradation. The highest concentration enhanced the expression of two efflux pump components, MdtE/F, highlighting the concentration-effect on the adaptive regulatory mechanisms. Although most of the changes were suggested to improve bacterial fitness against the effect of polymyxin B, it was also hypothesized that the upregulation of FadD could indirectly contribute to the membrane damage caused by the highest concentration due to excessive lipopolysaccharide biosynthesis [46].

Several studies have highlighted proteins and metabolic pathways involved in colistin resistance in bacterial species. Li et al. [43] studied changes in *mcr-1-*mediated colistin-resistant and sensitive *E. coli* using a label-free proteomic approach to understand the consecutive changes in colistin-resistant strain in presence of antibiotics. In this study, protein levels were significantly changed under selective pressure of both colistin and polymyxin B, and more specifically, out of the 14 differentially expressed proteins ydcH, osmY and tolA were suggested to play an important role in the *mcr-1* mediated colistin resistance in *E. coli*. Changes in the number of differentially expressed proteins located on the bacterial membrane were also reported when the bacteria had been exposed to different concentrations of colistin [43]. This highlights another important aspect of information that proteomics provides which is the antibiotic concentration-dependent response of bacteria. Bacterial adaptation to drug selection pressure and drug tolerance could be associated and investigated as a result of this kind of bacterial response. Two other studies have looked at the association between colistin resistance and molecular protein response in *A. baumanni*. Increased expression of the efflux pump system (Figure 1) and downregulation of two proteins, FabZ and beta-lactamase as a response to a loss of lipopolysaccharides that mediate colistin activity in colistin-resistant *A. baumannii* has been reported [65] (Table 2). In another study, *A. baumannii* adaptation to colistin treatment revealed 35 differentially expressed proteins in the colistin-resistant strain, most associated with the outer membrane [45]. As bacterial biofilms represent another clinically important phenotype, studies the efficacy of antibiotics on bacterial biofilms have also made use of proteomics. Pulsed-SILAC proteomic approach has been used to quantify proteins that are newly expressed in colistin-tolerant subpopulations in *P. aeruginosa* biofilms [66] (Table 2). Differentially expressed proteins with a focus on those associated with quorum sensing and migration had been studied in detail.

#### 2.4.2. Daptomycin

Daptomycin is a natural cyclic lipopeptide that is produced by *Streptomyces roseosporus* (Figure 6) [127]. It is used in for treatment of life-threatening infections caused by a wide range of Gram-positive bacteria. For example, it has antibacterial activity against vancomycin-resistant enterococci, MRSA, glycopeptide-intermediate *S. aureus*, and penicillin-resistant *S. pneumoniae* [128]. It is also frequently used for life-threatening septic pneumococcal disease [46] and treatment of severe *S. aureus* skin and soft tissue infections linked to endocarditis and staphylococcal sepsis [129]. Daptomycin’s antibacterial mode of action is still under investigation. Though there is evidence of a structural transition of daptomycin as it enters the cell membrane. The presence of Ca^2+^ ions and anionic phospholipid phosphatidylglycerol is particularly important for this to happen [46,130]. Interaction with Ca^2+^ can decrease the MIC of Daptomycin approximately 50-fold [131]. As with any antibiotic, resistance to daptomycin has been documented against, e.g., *S. aureus* and enterococci. The resistance mechanism includes modification of regulatory systems in the bacteria and enzymes involved in phospholipid metabolism [118]. Furthermore, quantitative proteomic approaches have investigated bacterial response on systemic protein level in response do daptomycin treatment. Antibiotics can affect the expression levels of distinct proteins. Proteomic analyses, for the first time, have been used to report on the differential protein expression in *S. aureus* in the presence of daptomycin. Here, seven bacterial cell membrane proteins had been altered and this change had been associated with the overall decrease of the bacterial membrane potential leading to bacterial membrane disintegration and, eventually, cell death [48]. Another study had reported on the change in protein levels during daptomycin resistance development so that the cellular changes in the presence of increasing concentrations of daptomycin could be elucidated [47].

### 2.5. Promising Drug Candidates: Antimicrobial Peptides

Antimicrobial peptides are a crucial component of the innate immune response and have a broad and diverse antibacterial, antifungal, antiviral, and even anticancer activity [132]. They are considered to be potent and highly-promising therapeutic agents because of the benefits that they provide over currently used antibiotics [133]. Their main advantage is attributed to their structural features; thus, it is noteworthy that there are striking structural overlaps between some antimicrobial peptides and some of the last resort antibiotics like polymyxin and daptomycin. Overall, antimicrobial peptides are relatively short, amphipathic, rich in cationic amino acids, and typically consist of less than 100 amino acids [134]. They are divided into four major categories regarding their secondary structure: α-helical, β-sheets, extended structures and di-sulfide bridge stabilized structures consisting of combinations of the above structural features [135]. The primary antibacterial mode of action was considered to be through electrostatic interaction with the anionic lipids of the outer bacterial membrane and membrane permeabilization [136]. There have been three established models describing this interaction, which are the pore-forming, the toroidal, and the carpet model [137]. It is currently known that these models cannot describe efficiently the action of all antimicrobial peptides. Several studies have demonstrated that although membrane association could be the first step for antimicrobial peptides to exert their function, it is not necessarily the main bacterial target, nor a sufficient mechanism to cause cell death alone [138]. There is evidence that antimicrobial peptides can have multiple intracellular targets as well, including DNA and protein synthesis inhibition, protein-folding interruption, interference with the activity of proteases, peptidoglycan biosynthesis, and endotoxin neutralization [139]. More evidence is required to be able to confidently predict the precise behavior of these agents in different environments and utilize their potential as novel antibiotics and more effort is needed to overcome the current difficulties they pose. LL-37 is a well-described cationic peptide that is capable of disrupting bacterial membranes, bind DNA [140], and target intercellular bacterial processes like production of reactive oxygen species [141]. A recent study had used high-resolution mass spectroscopy proteomic approaches to investigate the changes in *S. pneumoniae* in presence of LL-37 and the findings of this study show that several proteins could be involved in resistance development to LL-37 and many others that aid bacterial adaptation in presence of LL-37 [61] (Table 2). These observations could reflect the bacterial response towards other antimicrobial peptides that share the mode of action with LL-37.

## 3. Discussion and Conclusions

Harmful bacteria continue to pose a health threat on a global scale. Bacteria have developed multiple mechanisms to ensure their survival in the human host. At the same time, innovative ’omics’ technologies such as functional genomics, transcriptomics, metabolomics, and proteomics have also advanced, generating a plethora of material and methods that can assist the progress in clinical microbiology. These technologies continue to increase our overall understanding of the complexity of the microbial systems. Proteome analyses confirm the presence and quantity of proteins and allow for identification of a wide variety of chemical modifications found on proteins. A significant number of studies have already highlighted the importance of proteomics by creating protein profiles of different responses triggered by components of most antibiotics classes and providing a better way of tracking the process of infection based on these profiles. Determining total proteome alterations in strains that show different levels of resistance or different phenotypic reactions to antibiotics can be used in a comparative manner to fill in the gaps in the characterization of the different responses. In the efforts to combine the existing knowledge in this area of research we combined selected studies and summarized them with regards to the type of antibiotic used, pathogen, experimental conditions, proteomic approaches (Table 2). Diversity in the use of different approaches to investigate antibiotic responses can be noticed across the different studies and this highlights the complexity of proteomic investigations and the urge for more publications within this area. Even though there are multiple studies on the bacterial proteomes that increase our understanding of the underlying antibiotic mechanism of action and bacterial responses, proteomics investigations have yet to assist in development of novel antibiotic classes.

There are still challenges in using proteomics as a tool to identify and quantify proteins that need to be addressed. In bacteria, identification of low levels of modifications can be challenging and this differs based on the biological conditions. In parallel with the use of these advanced technologies, data processing and characterizations may differ as there is still room for development. An important factor to account for when starting a proteome analysis is the capabilities of the accessible mass spectrometer. Secondly, the scope of the project, sample amounts, mass spectrometry time available, budget for reagents, availability of proper bioinformatics analysis, etc., need to be considered.

In summary, with the increased understanding of the data analysis generated by different proteomic approaches, researchers are slowly starting to apply this technique in deciphering the proteome profiles in several bacterial species. Some of the important aspects where the scientific community will gain significant knowledge are the system level response of resistance and tolerance development in bacteria towards the last resort antibiotics. The new generated information will not only allow the discovery of unknown function of many proteins, but also improve the strategies for developing novel antimicrobial agents with improved bacterial targets. Knowledge in this area, especially in bacterial response to antibiotic treatments, is pivotal for improving our understanding of treatment failure and refine our treatment strategies.

## Figures and Tables

**Table 1:** Priority pathogens published by the World Health Organization (WHO) in 2017. The table summarizes the antibiotic classes and their mechanisms of resistance. * *Enterobacteriaceae* includes *K. pneumoniae*, *Escherichia coli*, *Serratia marcesens,* and *Proteus mirabilis*.

Pathogen	Resistance	Priority	Gram +/−
*Acinetobacter baumannii*	carbapenem-resistant	Critical	−
*Pseudomonas aeruginosa*	carbapenem-resistant	Critical	−
* *Enterobacteriaceae*	carbapenem-resistant, extended-spectrum β lactamase (ESBL)-producing	Critical	−
*Enterococcus faecium*	vancomycin-resistant	High	+
*Staphylococcus aureus*	methicillin-resistant,vancomycin-intermediate and resistant	High	+
*Helicobacter pylori*	clarithromycin-resistant	High	−
*Campylobacter spp.*	fluoroquinolone-resistant	High	−
*Salmonellae*	fluoroquinolone-resistant	High	−
*Neisseria gonorrhoeae*	cephalosporin-resistant, fluoroquinolone-resistant	High	−
*Streptococcus pneumoniae*	penicillin-non-susceptible	Medium	+
*Haemophilus influenzae*	ampicillin-resistant	Medium	−
*Shigella spp.*	fluoroquinolone-resistant	Medium	−

**Table 2:** Summary of selected literature, shown in descending order from 2020 to 2010, on proteomic analysis of antibiotic response in different bacteria.

Drug	Bacterial Strain	Primary MOA	Phenotypic Investigation, Target	Time of Exposure	Concentration	Proteomic Approach and Method	Proteome Coverage (%) *	Results, Selected DEPs	Reference
PMB	*E. coli*	Outer membrane	Planktonic, Adaptive responses, (tolerance)	30 growth cycles 31 × 16 h	1 µg/mL 5 µg/mL 10 µg/ml	iTRAQ labeling (TMT) LC-MS/MS	4,558 (pr. sample)	1 µg/mL: (66 ↑, 22 ↓) 10 µg/mL: (232 ↑, 82 ↓)	[46]
LL-37	*Strep. pneumoniae*	Bacterial membrane, DNA	Planktonic, resistance	1–2 h	2.5 µg/ml	Label-free, DDA	68%, 1,293/195	23-↑ 29-↓	[61]
CIP	*E. coli*	DNA gyrase	Persisters	3 h, cyclic antibiotic exposure	5 µg/mL (100 × MIC)	Label-free, DDA	739	23 ↑ 12 ↓	[62]
RIF AMP	*E. coli*	RNA synthesis, cell wall	Persisters	30 min RIF 3 h AMP	100 µg/mL RIF, 100 µg/mL AMP	Label-free, DDA	1,160	70 ↓ 35 ↑(persisters)	[59]
CST PMB	*E. coli*	Outer membrane	mcr-1 colistin-resistance	NS	0, 0.5–1 μg/mL	Label-free, DDA (TOF-MS/MS) DIA	64.3%, 2,784	No drug: 26 ↑, 31 ↓ CST 0.5: 75 ↑, 311 ↓ CST 1: 69 ↑, 237 ↓ PMB 0.5: 35 ↑, 219 ↓ PMB 1: 16 ↑, 147 ↓	[43]
TOB	*P. aeruginosa*	Protein synthesis	Outer membrane vesicles	24 h	1 µg/mL (sub-MIC)	Label-free, iBAQ,	757	165 ↓,17 ↑	[42]
-	*E. coli (ESBL-ST131)*	Cell wall biosynthesis	Characterization of clinical isolates	No exposure	-	MALDI-TOF MS, LC-MS/MS	10	-	[62]
AgNPs	*P. aeruginosa*	Cell membrane, ROS generation	Planktonic	24 h	0.1–50 µg/mL	Labeling, iTRAQ	ND	3-↑,5-supressed	[49]
AMP CTX CFP	*E. coli*	Cell wall biosynthesis	Outer membrane vesicles, β-lactam resistance	12–84 h	AMP: 30 µg/mL CTX: 4 µg/mL CFP: 1.25 µg/mL	SDS-PAGE, MALDI-TOF-MS	1,639 (273 mapped) 260 (OMVs resistant) 270 (OMVs susceptible)	83 ↑, 49 ↓ (resistant)	[63]
MEM CIP	*E. coli(NDM, KPC or IMP)*	Cell wall biosynthesis, DNA gyrase	Planktonic, β-lactam resistance	4 h	sub-MIC 0.3–24 µg/mL MEM 32 µg/mL CIP	DIA	457	OmpA: (all strains-CIP) ↓ and (IMP-MEM)↑ HU DNA-bp: (NDM and KPC-MEM) ↑ GroEL/GroES and GrpE: (all strains-MEM) ↑	[64]
DAP	*S. aureus*	Lipoteichoic acid biosynthesis	Planktonic, biofilm, resistant	4 months (Daily passages)	0–31 µg/mL	Labeling	60%, 1,709	349 DEPs, 105 ↑ 80 ↓	[47]
CIP	*P. aeruginosa*	DNA gyrase	Biofilms- antibiotic tolerant subpopulation	1.5, 5.5, 14.5 h	60 µg/Ml (Supra-MIC)	Label (BONCAT enrichment)	> 1,200	1.5h: 73 (41 unique) 5.5 h: 187 (80 unique) 14.5: 204 (90 unique)	[39]
CIP	*P. aeruginosa*	DNA gyrase	Planktonic, adaptive resistance	48 h	0.125–8 µg/mL	Label-free, DDA	57.6% 3,251	mu0125_l: 57 ↑,76 ↓ mu0125_h:62 ↑,92 ↓ mu05_l: 43 ↑, 26 ↓ mu05_h: 48 ↑, 68 ↓	[40]
DAP	*S. aureus*	Lipoteichoic acidbiosynthesis	Planktonic, resistant and sensitive population	18 h	0.25–2 × MIC	Labeling, iTRAQ	872	34 ↑ 17 ↓	[48]
CST	*A. baumannii MDR-ZJ06*	Outer membrane	Planktonic, resistance in MDR strains	ON cultures	8 × MIC 64 × MIC 200 × MIC	Labeling, iTRAQ	1,582	31 ↑ 51 ↓	[65]
CIP BC	*P. aeruginosa*	DNA gyrase, outer membrane	Outer membrane proteins, Biofilms, adaptive resistance	12 days	CIP: 6 µg/mL BC: 324 µg/mL	2-DE SDS-PAGE	10 proteins/600 spots	9 ↓ 1 ↑	[38]
CST	*P. aeruginosa*	Outer membrane	Biofilms, Tolerance	2-32 h, 8 h	10 µg/mL-(10 × MIC)	Label - Pulsed-SILAC	4,250	256 ↑ 140 ↓	[66]
OFX	*M. tuberculosis*	DNA gyrase	Planktonic, Mono-resistance	36 h	2 µg/mL (sub-MIC)	2-DE, MALDI-TOF MS	14	14 ↑	[67]
KAN	*E. coli*	Protein synthesis	Outer membrane Resistance	10 sequential subcultures	6.25 µg/mL (1/2 MIC)	2-DE, MALDI-TOF MS	11	6 ↑ 5 ↓	[68]
TOB	*P. aeruginosa*	Protein synthesis	Planktonic, adaptive resistance	DDE: 60 min TCE: 15, 60, 120, 360 min	DDE: 0.1, 0.5, 1µg/mL TCE: 1 µg/mL	DDA	> 1000 (TOB)	96 ↑	[41]
OXA	*MRSA, MSSA*	Cell wall biosynthesis	Planktonic	NS	sub-MIC 1/8 × MIC 8 µg/mL 0.125 µg/mL	Label-free, DDA	MRSA: 1,071, (41%) MSSA: 1,034 (40%)	MRSA: 65 ↑,16 ↓ MSSA: 162 ↑, 63 ↓	[69]
SM GEN CEF TET NA	*E. coli*	Protein synthesis, cell wall synthesis, DNA gyrase	Effect of low abundance of NarG and NarH on resistance	10 sequential subcultures	1/2 MIC	2-DE, MALDI-TOF MS	94	CAZ-R: 7↓ 6↑ SM-R: 5 ↓ 1↑ TET-R:7 ↓ 1↑ GEN-R: 9 ↓ 1↑ NA-R: 10 ↓	[70]
ERY	*E. coli*	Protein synthesis	Planktonic, resistance	0, 43, 68, 103 h	sub-MIC 10 µg/mL (sub-MIC)	2-DE, MALDI-TOF/TOF	35/91 sp.	43 h: 14 ↑, 3 ↓ 68 h: 6 ↓ 103h: 14 ↑, 1 ↓	[71]
CIP	*P. aeruginosa*	DNA gyrase	Planktonic, adaptive resistance	0–48 h	PAO1: 0–8 × MIC (0–4 µg/mL) PAK: 1/2 MIC (0.125 µg/mL)	2-DE, MALDI-TOF/TOF	3/650 sp.	2 proteins with higher phosphorylated/total protein ratio 1 ↑	[37]
β-lactams	*E. coli* *(TEM-52 and CMY-2)*	Cell wall biosynthesis	β-lactam resistance mediated	No exposure	-	2-DE, IEF, MALDI-TOF/TOF	C583 strain: 64 sp. C580 strain: 91 sp.	-	[72]

* Total number of proteins identified; CST-Colistin; PMB-Polymyxin B; CIP-Ciprofloxacin; TOB-Tobramycin; AMP-Ampicillin; BC-Benzalkonium Chloride; CAZ-ceftazidime, SM-streptomycin; ERY-erythromycin; GEN-gentamicin; CEF- ceftazidime; OFX-ofloxacin; OXA-oxacillin; DAP-daptomycin; MOA-Mode of action; arrow up-regulated and arrow down-downregulated proteins; SWATH -Sequential Window Acquisition of All Theoretical mass spectra method; IEF-isoelectric focusing; NS-not stated; ND-not determined; MIC-minimum inhibitory concentration; OMV-outer membrane vesicles; DDE-dose dependent experiment; TCE-time course experiment; bp-binding proteins; SDS-PAGE- Sodium Dodecyl Sulfate–Polyacrylamide Gel Electrophoresis; LC-MS- Liquid chromatography-mass spectrometry.

## References

[1] Prestinaci F., Pezzotti P., Pantosti A. (2015). Antimicrobial resistance: A global multifaceted phenomenon. Pathog. Glob. Health.

[2] Antibiotic resistance threats in the United States 2019. https://www.cdc.gov/drugresistance/pdf/threats-report/2019-ar-threats-report-508.pdf.

[3] Cassini A., Högberg L.D., Plachouras D., Quattrocchi A., Hoxha A., Simonsen G.S., Colomb-Cotinat M., Kretzschmar M.E., Devleesschauwer B., Cecchini M. (2019). Attributable deaths and disability-adjusted life-years caused by infections with antibiotic-resistant bacteria in the EU and the European Economic Area in 2015: A population-level modelling analysis. Lancet. Infect. Dis..

[4] The Review on Antimicrobial Resistance. https://Amr-Review.Org/.

[5] Conibear T.C.R., Collins S.L., Webb J.S. (2009). Role of Mutation in Pseudomonas aeruginosa Biofilm Development. PLoS ONE.

[6] Erickson B.E. (2001). Gene Transfer in the Environment. Environ. Sci. Technol..

[7] Kapoor G., Saigal S., Elongavan A. (2017). Action and resistance mechanisms of antibiotics: A guide for clinicians. J. Anaesthesiol. Clin. Pharmacol..

[8] Rodrigue A., Quentin Y., Lazdunski A., Méjean V., Foglino M. (2000). Cell signalling by oligosaccharides. Two-component systems in Pseudomonas aeruginosa: Why so many?. Trends Microbiol..

[9] Fernández L., Jenssen H., Bains M., Wiegand I., Gooderham W.J., Hancock R.E.W. (2012). The Two-Component System CprRS Senses Cationic Peptides and Triggers Adaptive Resistance in Pseudomonas aeruginosa Independently of ParRS. Antimicrob. Agents Chemother..

[10] Lewenza S. (2013). Extracellular DNA-induced antimicrobial peptide resistance mechanisms in Pseudomonas aeruginosa. Front. Microbiol..

[11] Yen M., Peabody C.R., Partovi S.M., Zhai Y., Tseng Y., Saier M.H. (2002). Protein-translocating outer membrane porins of Gram-negative bacteria. Biochim. Biophys. Acta (BBA) Biomembr..

[12] Kulkarni A.P., Nagvekar V.C., Veeraraghavan B., Warrier A.R., TS D., Ahdal J., Jain R. (2019). Current Perspectives on Treatment of Gram-Positive Infections in India: What Is the Way Forward?. Interdiscip. Perspect. Infect. Dis..

[13] Santajit S., Indrawattana N. (2016). Mechanisms of Antimicrobial Resistance in ESKAPE Pathogens. Biomed Res. Int..

[14] Founou R.C., Founou L.L., Essack S.Y. (2017). Clinical and economic impact of antibiotic resistance in developing countries: A systematic review and meta-analysis. PLoS One.

[15] LaFleur M.D., Qi Q., Lewis K. (2010). Patients with Long-Term Oral Carriage Harbor High-Persister Mutants of Candida albicans. Antimicrob. Agents Chemother..

[16] Bartell J.A., Cameron D.R., Mojsoska B., Haagensen J.A., Sommer L.M., Lewis K., Molin S., Johansen H.K. (2020). Bacterial persisters in long-term infection: Emergence and fitness in a complex host environment. Under Rev. (PLOS Pathog.).

[17] Mulcahy L.R., Burns J.L., Lory S., Lewis K. (2010). Emergence of Pseudomonas aeruginosa strains producing high levels of persister cells in patients with cystic fibrosis. J. Bacteriol..

[18] Balaban N.Q., Helaine S., Lewis K., Ackermann M., Aldridge B., Andersson D.I., Brynildsen M.P., Bumann D., Camilli A., Collins J.J. (2019). Definitions and guidelines for research on antibiotic persistence. Nat. Rev. Microbiol..

[19] Pu Y., Zhao Z., Li Y., Zou J., Ma Q., Zhao Y., Ke Y., Zhu Y., Chen H., Baker M.A.B. (2016). Enhanced Efflux Activity Facilitates Drug Tolerance in Dormant Bacterial Cells. Mol. Cell.

[20] Keren I., Shah D., Spoering A., Kaldalu N., Lewis K. (2004). Specialized Persister Cells and the Mechanism of Multidrug Tolerance in Escherichia coli. J. Bacteriol..

[21] Keren I., Minami S., Rubin E., Lewis K. (2011). Characterization and Transcriptome Analysis of Mycobacterium tuberculosis Persisters. MBio.

[22] Rowe S.E., Wagner N.J., Li L., Beam J.E., Wilkinson A.D., Radlinski L.C., Zhang Q., Miao E.A., Conlon B.P. (2020). Reactive oxygen species induce antibiotic tolerance during systemic Staphylococcus aureus infection. Nat. Microbiol..

[23] Kwan B.W., Valenta J.A., Benedik M.J., Wood T.K. (2013). Arrested Protein Synthesis Increases Persister-Like Cell Formation. Antimicrob. Agents Chemother..

[24] Macek B., Forchhammer K., Hardouin J., Weber-Ban E., Grangeasse C., Mijakovic I. (2019). Protein post-translational modifications in bacteria. Nat. Rev. Microbiol..

[25] Patel V.J., Thalassinos K., Slade S.E., Connolly J.B., Crombie A., Murrell J.C., Scrivens J.H. (2009). A Comparison of Labeling and Label-Free Mass Spectrometry-Based Proteomics Approaches. J. Proteome Res..

[26] Ong S.-E., Blagoev B., Kratchmarova I., Kristensen D.B., Steen H., Pandey A., Mann M. (2002). Stable isotope labeling by amino acids in cell culture, SILAC, as a simple and accurate approach to expression proteomics. Mol. Cell. Proteomics.

[27] Han J., Yi S., Zhao X., Zheng Y., Yang D., Du G., Yang X.-Y., He Q.-Y., Sun X. (2019). Improved SILAC method for double labeling of bacterial proteome. J. Proteomics.

[28] Ross P.L., Huang Y.N., Marchese J.N., Williamson B., Parker K., Hattan S., Khainovski N., Pillai S., Dey S., Daniels S. (2004). Multiplexed Protein Quantitation in Saccharomyces cerevisiae Using Amine-reactive Isobaric Tagging Reagents. Mol. Cell. Proteomics.

[29] Thompson A., Schäfer J., Kuhn K., Kienle S., Schwarz J., Schmidt G., Neumann T., Hamon C. (2003). Tandem Mass Tags: A Novel Quantification Strategy for Comparative Analysis of Complex Protein Mixtures by MS/MS. Anal. Chem..

[30] Pérez-Llarena F.J., Bou G. (2016). Proteomics As a Tool for Studying Bacterial Virulence and Antimicrobial Resistance. Front. Microbiol..

[31] Sulaiman J.E., Lam H. (2019). Application of proteomics in studying bacterial persistence. Expert Rev. Proteomics.

[32] Yung Y.P., McGill S.L., Chen H., Park H., Carlson R.P., Hanley L. (2019). Reverse diauxie phenotype in Pseudomonas aeruginosa biofilm revealed by exometabolomics and label-free proteomics. NPJ Biofilms Microbiomes.

[33] Fagerquist C.K., Zaragoza W.J., Sultan O., Woo N., Quinones B., Cooley M.B., Mandrell R.E. (2014). Top-Down Proteomic Identification of Shiga Toxin 2 Subtypes from Shiga Toxin-Producing Escherichia coli by Matrix-Assisted Laser Desorption Ionization-Tandem Time of Flight Mass Spectrometry. Appl. Environ. Microbiol..

[34] Liu X., Gao B., Novik V., Galán J.E. (2012). Quantitative Proteomics of Intracellular Campylobacter jejuni Reveals Metabolic Reprogramming. PLoS Pathog..

[35] Vorwerk S., Krieger V., Deiwick J., Hensel M., Hansmeier N. (2015). Proteomes of host cell membranes modified by intracellular activities of Salmonella enterica. Mol. Cell. Proteomics.

[36] Zhou J., Hao D., Wang X., Liu T., He C., Xie F., Sun Y., Zhang J. (2006). An important role of a “probable ATP-binding component of ABC transporter” during the process of Pseudomonas aeruginosa resistance to fluoroquinolone. Proteomics.

[37] Su H.-C., Ramkissoon K., Doolittle J., Clark M., Khatun J., Secrest A., Wolfgang M.C., Giddings M.C. (2010). The Development of Ciprofloxacin Resistance in Pseudomonas aeruginosa Involves Multiple Response Stages and Multiple Proteins. Antimicrob. Agents Chemother..

[38] Machado I., Coquet L. (2016). Proteomic Changes in Pseudomonas aeruginosa Biofilm Cells after Adaptive Resistance Development. J. Proteomics Bioinform..

[39] Babin B.M., Atangcho L., van Eldijk M.B., Sweredoski M.J., Moradian A., Hess S., Tolker-Nielsen T., Newman D.K., Tirrell D.A. (2017). Selective Proteomic Analysis of Antibiotic-Tolerant Cellular Subpopulations in Pseudomonas aeruginosa Biofilms. MBio.

[40] Peng J., Cao J., Ng F.M., Hill J. (2017). Pseudomonas aeruginosa develops Ciprofloxacin resistance from low to high level with distinctive proteome changes. J. Proteomics.

[41] Wu X., Held K., Zheng C., Staudinger B.J., Chavez J.D., Weisbrod C.R., Eng J.K., Singh P.K., Manoil C., Bruce J.E. (2015). Dynamic Proteome Response of Pseudomonas aeruginosa to Tobramycin Antibiotic Treatment. Mol. Cell. Proteomics.

[42] Koeppen K., Barnaby R., Jackson A.A., Gerber S.A., Hogan D.A., Stanton B.A. (2019). Tobramycin reduces key virulence determinants in the proteome of Pseudomonas aeruginosa outer membrane vesicles. PLoS One.

[43] LeBel M. (1988). Ciprofloxacin: Chemistry, mechanism of action, resistance, antimicrobial spectrum, pharmacokinetics, clinical trials, and adverse reactions. Pharmacotherapy.

[44] Moffatt J.H., Harper M., Harrison P., Hale J.D.F., Vinogradov E., Seemann T., Henry R., Crane B., St. Michael F., Cox A.D. (2010). Colistin Resistance in Acinetobacter baumannii Is Mediated by Complete Loss of Lipopolysaccharide Production. Antimicrob. Agents Chemother..

[45] Fernández-Reyes M., Rodríguez-Falcón M., Chiva C., Pachón J., Andreu D., Rivas L. (2009). The cost of resistance to colistin in Acinetobacter baumannii: A proteomic perspective. Proteomics.

[46] Henken S., Bohling J., Martens-Lobenhoffer J., Paton J.C., Ogunniyi A.D., Briles D.E., Salisbury V.C., Wedekind D., Bode-Böger S.M., Welsh T. (2010). Efficacy profiles of daptomycin for treatment of invasive and noninvasive pulmonary infections with Streptococcus pneumoniae. Antimicrob. Agents Chemother..

[47] Müller A., Grein F., Otto A., Gries K., Orlov D., Zarubaev V., Girard M., Sher X., Shamova O., Roemer T. (2018). Differential daptomycin resistance development in Staphylococcus aureus strains with active and mutated gra regulatory systems. Int. J. Med. Microbiol..

[48] Ma W., Zhang D., Li G., Liu J., He G., Zhang P., Yang L., Zhu H., Xu N., Liang S. (2017). Antibacterial mechanism of daptomycin antibiotic against Staphylococcus aureus based on a quantitative bacterial proteome analysis. J. Proteomics.

[49] Yan X., He B., Liu L., Qu G., Shi J., Hu L., Jiang G. (2018). Antibacterial mechanism of silver nanoparticles in Pseudomonas aeruginosa: Proteomics approach. Metallomics.

[50] Bateman A., Martin M.J., O’Donovan C., Magrane M., Apweiler R., Alpi E., Antunes R., Arganiska J., Bely B., Bingley M. (2015). UniProt: A hub for protein information. Nucleic Acids Res..

[51] Gaviard C., Jouenne T., Hardouin J. (2018). Proteomics of Pseudomonas aeruginosa: The increasing role of post-translational modifications. Expert Rev. Proteomics.

[52] Boucher R.C. (2007). Cystic fibrosis: A disease of vulnerability to airway surface dehydration. Trends Mol. Med..

[53] Fujitani S., Sun H.-Y., Yu V.L., Weingarten J.A. (2011). Pneumonia Due to Pseudomonas aeruginosa. Chest.

[54] Rakhimova E., Wiehlmann L., Brauer A.L., Sethi S., Murphy T.F., Tümmler B. (2009). Pseudomonas aeruginosa Population Biology in Chronic Obstructive Pulmonary Disease. J. Infect. Dis..

[55] Ouidir T., Jouenne T., Hardouin J. (2016). Post-translational modifications in Pseudomonas aeruginosa revolutionized by proteomic analysis. Biochimie.

[56] Wu X., Siehnel R.J., Garudathri J., Staudinger B.J., Hisert K.B., Ozer E.A., Hauser A.R., Eng J.K., Manoil C., Singh P.K. (2019). In Vivo Proteome of Pseudomonas aeruginosa in Airways of Cystic Fibrosis Patients. J. Proteome Res..

[57] Ouidir T., Cosette P., Jouenne T., Hardouin J. (2015). Proteomic profiling of lysine acetylation in Pseudomonas aeruginosa reveals the diversity of acetylated proteins. Proteomics.

[58] Schmidt A., Kochanowski K., Vedelaar S., Ahrné E., Volkmer B., Callipo L., Knoops K., Bauer M., Aebersold R., Heinemann M. (2016). The quantitative and condition-dependent Escherichia coli proteome. Nat. Biotechnol..

[59] Sulaiman J.E., Hao C., Lam H. (2018). Specific Enrichment and Proteomics Analysis of Escherichia coli Persisters from Rifampin Pretreatment. J. Proteome Res..

[60] Sulaiman J.E., Lam H. (2020). Proteomic Investigation of Tolerant Escherichia coli Populations from Cyclic Antibiotic Treatment. J. Proteome Res..

[61] Mücke P.-A., Maaß S., Kohler T.P., Hammerschmidt S., Becher D. (2020). Proteomic Adaptation of Streptococcus pneumoniae to the Human Antimicrobial Peptide LL-37. Microorganisms.

[62] Nakamura A., Komatsu M., Ohno Y., Noguchi N., Kondo A., Hatano N. (2019). Identification of specific protein amino acid substitutions of extended-spectrum β-lactamase (ESBL)-producing Escherichia coli ST131: A proteomics approach using mass spectrometry. Sci. Rep..

[63] Kim S.W., Park S.B., Im S.P., Lee J.S., Jung J.W., Gong T.W., Lazarte J.M.S., Kim J., Seo J.-S., Kim J.-H. (2018). Outer membrane vesicles from β-lactam-resistant Escherichia coli enable the survival of β-lactam-susceptible E. coli in the presence of β-lactam antibiotics. Sci. Rep..

[64] Sidjabat H.E., Gien J., Kvaskoff D., Ashman K., Vaswani K., Reed S., McGeary R.P., Paterson D.L., Bordin A., Schenk G. (2018). The use of SWATH to analyse the dynamic changes of bacterial proteome of carbapanemase-producing Escherichia coli under antibiotic pressure. Sci. Rep..

[65] Hua X., Liu L., Fang Y., Shi Q., Li X., Chen Q., Shi K., Jiang Y., Zhou H., Yu Y. (2017). Colistin Resistance in Acinetobacter baumannii MDR-ZJ06 Revealed by a Multiomics Approach. Front. Cell. Infect. Microbiol..

[66] Chua S.L., Yam J.K.H., Hao P., Adav S.S., Salido M.M., Liu Y., Givskov M., Sze S.K., Tolker-Nielsen T., Yang L. (2016). Selective labelling and eradication of antibiotic-tolerant bacterial populations in Pseudomonas aeruginosa biofilms. Nat. Commun..

[67] Lata M., Sharma D., Deo N., Tiwari P.K., Bisht D., Venkatesan K. (2015). Proteomic analysis of ofloxacin-mono resistant Mycobacterium tuberculosis isolates. J. Proteomics.

[68] Zhang D., Li H., Lin X., Peng X. (2015). Outer membrane proteomics of kanamycin-resistant Escherichia coli identified MipA as a novel antibiotic resistance-related protein. FEMS Microbiol. Lett..

[69] Liu X., Hu Y., Pai P.-J., Chen D., Lam H. (2014). Label-Free Quantitative Proteomics Analysis of Antibiotic Response in Staphylococcus aureus to Oxacillin. J. Proteome Res..

[70] Ma Y., Guo C., Li H., Peng X.X. (2013). Low abundance of respiratory nitrate reductase is essential for Escherichia coli in resistance to aminoglycoside and cephalosporin. J. Proteomics.

[71] Petrackova D., Janecek J., Bezouskova S., Kalachova L., Technikova Z., Buriankova K., Halada P., Haladova K., Weiser J. (2013). Fitness and proteome changes accompanying the development of erythromycin resistance in a population of Escherichia coli grown in continuous culture. Microbiologyopen.

[72] Pinto L., Poeta P., Radhouani H., Coelho C., Carvalho C., Rodrigues J., Torres C., Vitorino R., Domingues P., Igrejas G. (2011). Proteomic evaluation of escherichia coli isolates from human clinical strains. J. Integr. OMICS.

[73] Bush K., Bradford P.A. (2016). β-Lactams and β-Lactamase Inhibitors: An Overview. Cold Spring Harb. Perspect. Med..

[74] Wivagg C.N., Bhattacharyya R.P., Hung D.T. (2014). Mechanisms of β-lactam killing and resistance in the context of Mycobacterium tuberculosis. J. Antibiot. (Tokyo).

[75] Tomasz A. (1979). The Mechanism of the Irreversible Antimicrobial Effects of Penicillins: How the Beta-Lactam Antibiotics Kill and Lyse Bacteria. Annu. Rev. Microbiol..

[76] Tipper D.J. (1979). Mode of action of β-lactam antibiotics. Rev. Infect. Dis..

[77] Cho H., Uehara T., Bernhardt T.G. (2014). Beta-lactam antibiotics induce a lethal malfustioning of the bacterial cell wall sunthesis machinery. Cell.

[78] Xu C., Lin X., Ren H., Zhang Y., Wang S., Peng X. (2006). Analysis of outer membrane proteome of Escherichia coli related to resistance to ampicillin and tetracycline. Proteomics.

[79] Krause K.M., Serio A.W., Kane T.R., Connolly L.E. (2016). Aminoglycosides: An overview. Cold Spring Harb. Perspect. Med..

[80] Hancock R.E.W., Farmer S.W., Li Z., Poolet K. (1991). Interaction of Aminoglycosides with the Outer Membranes and Purified Lipopolysaccharide and OmpF Porin of Escherichia coli. Antimicrob. Agents Chemother..

[81] Taber H.W., Mueller J.P., Miller P.F., Arrow A.M.Y.S. (1987). Bacterial Uptake of Aminoglycoside Antibiotics. J. Antimicrob. Chemother..

[82] Jana S., Deb J.K. (2006). Molecular understanding of aminoglycoside action and resistance. Appl. Microbiol. Biotechnol..

[83] Ramirez M.S., Tolmasky M.E. (2010). Aminoglycoside Modifying Enzymes. Drug Resist. Updat..

[84] Tsai A., Uemura S., Johansson M., Puglisi E.V., Marshall R.A., Aitken C.E., Korlach J., Ehrenberg M., Puglisi J.D. (2013). The Impact of Aminoglycosides on the Dynamics of Translation Elongation. Cell Rep..

[85] Davis B.D., Chen L., Tai P.C. (1986). Misread protein creates membrane channels: An essential step in the bactericidal action of aminoglycosides. Proc. Natl. Acad. Sci. USA.

[86] Serio A.W., Keepers T., Andrews L., Krause K.M. (2018). Aminoglycoside Revival: Review of a Historically Important Class of Antimicrobials Undergoing Rejuvenation. EcoSal Plus.

[87] Shteinberg M., Elborn J.S. (2015). Use of Inhaled Tobramycin in Cystic Fibrosis. Adv. Ther..

[88] Bulitta J.B., Ly N.S., Landersdorfer C.B., Wanigaratne N.A., Velkov T., Yadav R., Oliver A., Martin L., Shin B.S., Forrest A. (2015). Two mechanisms of killing of pseudomonas aeruginosa by tobramycin assessed at multiple inocula via mechanism-based modeling. Antimicrob. Agents Chemother..

[89] Yadav R., Bulitta J.B., Schneider E.K., Shin B.S., Velkov T., Nation R.L., Landersdorfer C.B. (2017). Aminoglycoside Concentrations Required for Synergy with Carbapenems against Pseudomonas aeruginosa Determined via Mechanistic Studies and Modeling. Antimicrob. Agents Chemother..

[90] Park A.J., Murphy K., Surette M.D., Bandoro C., Krieger J.R., Taylor P., Khursigara C.M. (2015). Tracking the Dynamic Relationship between Cellular Systems and Extracellular Subproteomes in Pseudomonas aeruginosa Biofilms. J. Proteome Res..

[91] Reales-Calderón J.A., Corona F., Monteoliva L., Gil C., Martínez J.L. (2015). Quantitative proteomics unravels that the post-transcriptional regulator Crc modulates the generation of vesicles and secreted virulence determinants of Pseudomonas aeruginosa. J. Proteomics.

[92] Toyofuku M., Roschitzki B., Riedel K., Eberl L. (2012). Identification of Proteins Associated with the Pseudomonas aeruginosa Biofilm Extracellular Matrix. J. Proteome Res..

[93] Choi D.-S., Kim D.-K., Choi S.J., Lee J., Choi J.-P., Rho S., Park S.-H., Kim Y.-K., Hwang D., Gho Y.S. (2011). Proteomic analysis of outer membrane vesicles derived from Pseudomonas aeruginosa. Proteomics.

[94] Li H., Wang B.C., Xu W.J., Lin X.M., Peng X.X. (2008). Identification and network of outer membrane proteins regulating streptomysin resistance in escherichia coll. J. Proteome Res..

[95] Ćudić E., Surmann K., Panasia G., Hammer E., Hunke S. (2017). The role of the two-component systems Cpx and Arc in protein alterations upon gentamicin treatment in Escherichia coli. BMC Microbiol..

[96] Dinos G.P. (2017). The macrolide antibiotic renaissance. Br. J. Pharmacol..

[97] Janas A., Przybylski P. (2019). 14- and 15-membered lactone macrolides and their analogues and hybrids: Structure, molecular mechanism of action and biological activity. Eur. J. Med. Chem..

[98] Vázquez-Laslop N., Mankin A.S. (2018). How Macrolide Antibiotics Work. Trends Biochem. Sci..

[99] Svetlov M.S., Vázquez-Laslop N., Mankin A.S. (2017). Kinetics of drug-ribosome interactions defines the cidality of macrolide antibiotics. Proc. Natl. Acad. Sci. USA.

[100] Sothiselvam S., Neuner S., Rigger L., Klepacki D., Micura R., Vázquez-Laslop N., Mankin A.S. (2016). Binding of Macrolide Antibiotics Leads to Ribosomal Selection against Specific Substrates Based on Their Charge and Size. Cell Rep..

[101] Davis A.R., Gohara D.W., Yap M.-N.F. (2014). Sequence selectivity of macrolide-induced translational attenuation. Proc. Natl. Acad. Sci. USA.

[102] Yao W., Xu G., Li D., Bai B., Wang H., Cheng H., Zheng J., Sun X., Lin Z., Deng Q. (2019). Staphylococcus aureus with an erm-mediated constitutive macrolide-lincosamide-streptogramin B resistance phenotype has reduced susceptibility to the new ketolide, solithromycin. BMC Infect. Dis..

[103] Schroeder M.R., Stephens D.S. (2016). Macrolide Resistance in Streptococcus pneumoniae. Front. Cell. Infect. Microbiol..

[104] Gomes C., Ruiz-Roldán L., Mateu J., Ochoa T.J., Ruiz J. (2019). Azithromycin resistance levels and mechanisms in Escherichia coli. Sci. Rep..

[105] Fyfe C., Grossman T.H., Kerstein K., Sutcliffe J. (2016). Resistance to Macrolide Antibiotics in Public Health Pathogens. Cold Spring Harb. Perspect. Med..

[106] Chancey S.T., Zhou X., Zähner D., Stephens D.S. (2011). Induction of efflux-mediated macrolide resistance in Streptococcus pneumoniae. Antimicrob. Agents Chemother..

[107] Iannelli F., Santoro F., Santagati M., Docquier J.-D., Lazzeri E., Pastore G., Cassone M., Oggioni M.R., Rossolini G.M., Stefani S. (2018). Type M Resistance to Macrolides Is Due to a Two-Gene Efflux Transport System of the ATP-Binding Cassette (ABC) Superfamily. Front. Microbiol..

[108] Chollet R., Chevalier J., Bryskier A., Pagès J.-M. (2004). The AcrAB-TolC pump is involved in macrolide resistance but not in telithromycin efflux in Enterobacter aerogenes and Escherichia coli. Antimicrob. Agents Chemother..

[109] Andersson M.I. (2003). Development of the quinolones. J. Antimicrob. Chemother..

[110] Rehman A., Patrick W.M., Lamont I.L. (2019). Mechanisms of ciprofloxacin resistance in pseudomonas aeruginosa: New approaches to an old problem. J. Med. Microbiol..

[111] Naqvi S.A.R., Roohi S., Iqbal A., Sherazi T.A., Zahoor A.F., Imran M. (2018). Ciprofloxacin: From infection therapy to molecular imaging. Mol. Biol. Rep..

[112] Zhang G.F., Liu X., Zhang S., Pan B., Liu M.L. (2018). Ciprofloxacin derivatives and their antibacterial activities. Eur. J. Med. Chem..

[113] Palumbo M., Gatto B., Zagotto G., Palu G. (1993). On the mechanism of action of quinolone drugs. Bull. Johns Hopkins Hosp..

[114] Drlica K., Zhao X. (1997). DNA gyrase, topoisomerase IV, and the 4-quinolones. Microbiol. Mol. Biol. Rev..

[115] Jensen P.Ø., Briales A., Brochmann R.P., Wang H., Kragh K.N., Kolpen M., Hempel C., Bjarnsholt T., Høiby N., Ciofu O. (2014). Formation of hydroxyl radicals contributes to the bactericidal activity of ciprofloxacin against Pseudomonas aeruginosa biofilms. Pathog. Dis..

[116] Namvar A.E., Bastarahang S., Abbasi N., Ghehi G.S., Farhadbakhtiarian S., Arezi P., Hosseini M., Baravati S.Z., Jokar Z., Chermahin S.G. (2014). Clinical characteristics of Staphylococcus epidermidis: A systematic review. GMS Hyg. Infect. Control.

[117] Poirel L., Jayol A., Nordmann P. (2017). Polymyxins: Antibacterial Activity, Susceptibility Testing, and Resistance Mechanisms Encoded by Plasmids or Chromosomes. Clin. Microbiol. Rev..

[118] Miller W.R., Bayer A.S., Arias C.A. (2016). Mechanism of Action and Resistance to Daptomycin in Staphylococcus aureus and Enterococci. Cold Spring Harb. Perspect. Med..

[119] Li J., Nation R.L., Turnidge J.D., Milne R.W., Coulthard K., Rayner C.R., Paterson D.L. (2006). Colistin: The re-emerging antibiotic for multidrug-resistant Gram-negative bacterial infections. Lancet Infect. Dis..

[120] KOCH-WESER J. (1970). Adverse Effects of Sodium Colistimethate. Ann. Intern. Med..

[121] Moore R.A., Bates N.C., Hancock R.E.W. (1986). Interaction of polycationic antibiotics with Pseudomonas aeruginosa lipopolysaccharide and lipid A studied by using dansyl-polymyxin. Antimicrob. Agents Chemother..

[122] Hancock R.E.W. (1984). Alterations in Outer Membrane Permeability. Annu. Rev. Microbiol..

[123] Klemperer R.M., Gilbert P., Meier A.M., Cozens R.M., Brown M.R.W. (1979). Influence of Suspending Media upon the Susceptibility of Pseudomonas aeruginosa NCTC 6750 and Its Spheroplasts to Polymyxin B. Antimicrob. Agents Chemother..

[124] Zhang L., Dhillon P., Yan H., Farmer S., Hancock R.E.W. (2000). Interactions of Bacterial Cationic Peptide Antibiotics with Outer and Cytoplasmic Membranes of Pseudomonas aeruginosa. Antimicrob. Agents Chemother..

[125] Aquilini E., Merino S., Knirel Y., Regué M., Tomás J. (2014). Functional Identification of Proteus mirabilis eptC Gene Encoding a Core Lipopolysaccharide Phosphoethanolamine Transferase. Int. J. Mol. Sci..

[126] Anandan A., Evans G.L., Condic-Jurkic K., O’Mara M.L., John C.M., Phillips N.J., Jarvis G.A., Wills S.S., Stubbs K.A., Moraes I. (2017). Structure of a lipid A phosphoethanolamine transferase suggests how conformational changes govern substrate binding. Proc. Natl. Acad. Sci. USA.

[127] Li L., Ma T., Liu Q., Huang Y., Hu C., Liao G. (2013). Improvement of Daptomycin Production in Streptomyces roseosporus through the Acquisition of Pleuromutilin Resistance. Biomed Res. Int..

[128] Tally F.P., DeBruin M.F. (2000). Development of daptomycin for Gram-positive infections. J. Antimicrob. Chemother..

[129] Eisenstein B.I., Oleson F.B., Baltz R.H. (2010). Daptomycin: From the Mountain to the Clinic, with Essential Help from Francis Tally, MD. Clin. Infect. Dis..

[130] Jung D., Rozek A., Okon M., Hancock R.E.W. (2004). Structural Transitions as Determinants of the Action of the Calcium-Dependent Antibiotic Daptomycin. Chem. Biol..

[131] Chen Y.F., Sun T.L., Sun Y., Huang H.W. (2014). Interaction of daptomycin with lipid bilayers: A lipid extracting effect. Biochemistry.

[132] Seyfi R., Kahaki F.A., Ebrahimi T., Montazersaheb S., Eyvazi S., Babaeipour V., Tarhriz V. (2019). Antimicrobial Peptides (AMPs): Roles, Functions and Mechanism of Action. Int. J. Pept. Res. Ther..

[133] Raheem N., Straus S.K. (2019). Mechanisms of Action for Antimicrobial Peptides With Antibacterial and Antibiofilm Functions. Front. Microbiol..

[134] Mojsoska B., Jenssen H. (2015). Peptides and peptidomimetics for antimicrobial drug design. Pharmaceuticals.

[135] Lee T.-H.N., Hall K., Aguilar M.-I. (2015). Antimicrobial Peptide Structure and Mechanism of Action: A Focus on the Role of Membrane Structure. Curr. Top. Med. Chem..

[136] Lei J., Sun L.C., Huang S., Zhu C., Li P., He J., Mackey V., Coy D.H., He Q.Y. (2019). The antimicrobial peptides and their potential clinical applications. Am. J. Transl. Res..

[137] Brogden K.A. (2005). Antimicrobial peptides: Pore formers or metabolic inhibitors in bacteria?. Nat. Rev. Microbiol..

[138] Nicolas P. (2009). Multifunctional host defense peptides: Intracellular-targeting antimicrobial peptides. FEBS J..

[139] Le C.-F., Fang C.-M., Sekaran D. (2017). Intracellular Targeting Mechanisms by Antimicrobial Peptides. Antimicrob. Agents Chemother..

[140] Zhu Y., Mohapatra S., Weisshaar J.C. (2019). Rigidification of the Escherichia coli cytoplasm by the human antimicrobial peptide LL-37 revealed by superresolution fluorescence microscopy. Proc. Natl. Acad. Sci. USA.

[141] Liu W., Dong S.L., Xu F., Wang X.Q., Withers T.R., Yu H.D., Wang X. (2013). Effect of intracellular expression of antimicrobial peptide LL-37 on growth of escherichia coli strain TOP10 under aerobic and anaerobic conditions. Antimicrob. Agents Chemother..

